# “*The peace that I wanted*, *I got*”: Qualitative insights from patient experiences of SMART DAPPER interventions for major depression and traumatic stress disorders in Kenya

**DOI:** 10.1371/journal.pgph.0002685

**Published:** 2024-09-05

**Authors:** Monica Getahun, Muthoni A. Mathai, Grace Rota, Ammon Allen, Rachel L. Burger, Elizabeth Opiyo, Dennis Oluoch, Josyline Wangia, Raphael Wambura, Anne Mbwayo, Peter Muchembre, Raymond R. Obura, Thomas C. Neylan, Gregory A. Aarons, Linnet Ongeri, Susan M. Meffert

**Affiliations:** 1 Institute for Global Health Sciences, University of California San Francisco, San Francisco, California, United States of America; 2 Department of Psychiatry, University of Nairobi, Nairobi, Kenya; 3 Department of Psychiatry and Behavioral Sciences, University of California San Francisco, San Francisco, California, United States of America; 4 Global Programs for Research and Training, Nairobi, Kenya; 5 Kisumu County Hospital Psychiatric Unit, Kisumu, Kenya; 6 Department of Psychiatry, University of California San Diego, San Diego, California, United States of America; 7 Centre for Clinical Research, Kenya Medical Research Institute, Nairobi, Kenya; Dalhousie University, CANADA

## Abstract

SMART DAPPER is an implementation science study responding to mental health treatment gaps for depression and trauma-related disorders in Sub-Saharan Africa (SSA). We report on patient experiences in a study using a Sequential, Multiple Assignment Randomized Trial (SMART) design to test first and second line non-specialist treatment using psychotherapy (Interpersonal Psychotherapy [IPT] or medication (fluoxetine [FLX]), integrated within public sector primary care in western Kenya. An embedded qualitative study conducted in-depth interviews (n = 17) and three (n = 3) focus group discussions with participants (May to October 2021). Audio-recorded interviews were transcribed and translated into English; we deductively and inductively analyzed transcripts guided by grounded theoretical approaches and content analysis. We drew on the health belief model and socio-ecological framework to present findings, including perceived severity (motivations for taking part in the intervention), impacts of the intervention at the individual, interpersonal, and community and health systems levels as well as barriers and facilitators. Participants discussed family and marital conflict, loss of a child, loss of income or a job, and traumatic events such as a death or illness. Impacts at the individual level included reduced headaches, improved appetite and weight management, increased energy, improved sleep, better self-efficacy, and improved concentration, which was reported to lead to increased economic opportunities. At the interpersonal level, participants noted a reduction in conflict, better conflict management and resolution, increased harmony with family and community members, and improved relationships with their partners and children. Perceived challenges included balancing the intervention with livelihoods, preference for traditional medicines, actual or anticipated side effects with medication (FLX), mental health stigma, major life events, and perceived inadequate counseling and challenges with providers. The findings demonstrate the potential of the SMART DAPPER intervention for depression and trauma-related disorder treatments and underscore the challenges and barriers that must be addressed when scaling similar interventions.

**Trial registration: ClinicalTrials.gov identifier:** NCT03466346.

## Background

Mental disorders are a leading cause of global disability [1, 2], largely driven by depression and anxiety [3, 4]. Most of the disease burden is in Low and Middle Income Countries (LMICs), where approximately 75% of adults with mental disorders have no access to care—reflective of the widespread and persistent shortages in trained mental health professionals [5–7]. In Sub-Saharan Africa (SSA), many face challenges in accessing treatment due to complex and multifaceted challenges at the individual, health systems, and community levels [5–10]. The burden of mental health illness and unmet need is even higher among vulnerable populations including people living with HIV (PLHIV) [11–15]. In SSA, a region where an estimated 67% of PLHIV reside [16], rates of depression and post-traumatic stress disorder (PTSD) are elevated, reflective of the complex stigma and vulnerabilities that PLHIV experience [12–15]. Thus, addressing unmet mental health needs, especially among vulnerable populations including PLHIV, is critical public health priority [17, 18].

Some efforts are underway to address this burden of mental health illness in the region, including increased training, capacity building, and task shifting of mental health services [19–23]. Interpersonal therapy (IPT), for example, is increasingly used to address depression and PTSD. Studies have underscored the potential of IPT, including non-specialist delivered therapy, to address complex social cultural and personal dynamics which contribute to negative health outcomes [21, 24–30]. And yet, despite nearly 15 years of efficacy research showing that local non-specialists can provide evidence-based care for depression and anxiety in LMICs [31–36], few studies have advanced to the critical next step and morbidity from mental disorders continues to escalate [1, 37–39]. Further, not enough is known about how to implement non-specialist treatments at scale [38–41]. It is vital that global mental health treatment researchers now focus on implementation science to inform scale-up of evidence-based care to lower mental health burden. As emphasized by a recent World Health Organization (WHO) initiative [42], integration of mental health treatment into existing systems of care is critical to achieving public health impact.

In Kenya, specifically, the high prevalence of Major Depressive Disorder (MDD) (26%) and Posttraumatic Stress Disorder (PTSD) (35%) in primary care populations has led to their prioritization in treatment [43, 44]. Depression and trauma-related disorders are frequently co-morbid [45–47], and scalable treatment strategies in resource-constrained settings must be capable of treating both conditions using a non-specialist workforce. Kenyan healthcare providers and policy-makers collaborated to pioneer an innovative, government-funded initiative to scale-up treatment for mental disorders in primary healthcare [44, 48–50]. Data to guide the related scale-up of two essential treatments for depression and PTSD–psychotherapy and second generation antidepressants [51]—can thus inform Kenyan policy makers’ efforts to improve public sector mental health in the region.

SMART DAPPER provides data to inform evidence-based scale up of non-specialist delivery of treatment for depression and trauma-disorder, a much-needed approach with high potential to address mental health treatment gaps [52, 53], led by a team of investigators from several institutions and ministries of health in Kenya, Uganda, and the US. The project partners, together with local, national, and regional mental health stakeholders, evaluated non-specialist delivery of evidence-based depression and PTSD treatment, integrated within existing healthcare centers for “real world” non-specialist treatment to reduce population-level disability caused by depression and PTSD- priority conditions given their prevalence in Kenya. We formed a collaboration with KCRH and Kenyan national and county Ministry of Health (MOH) offices to identify scalable treatment interventions for this implementation research, with two key criteria: (1) interventions had to be first line treatment for both depression and PTSD with strong efficacy data for non-specialist delivery; and (2) had to meet current, global standard of care recommendations. The majority of mental health treatment studies in LMICs test psychotherapy/psychological interventions only, excluding pharmacological strategies. Yet, in many LMICs, psychotherapy and psychopharmacology (Selective-Serotonin Reuptake Inhibiters [SSRIs]) are considered the two essential pillars of first line treatment for depression and PTSD [54, 55].

SMART DAPPER tested access to *both* protocol driven psychotherapy and a modern SSRE, fluoxetine. The service-user acceptability of this treatment implementation strategy is crucial to informing scale up of similar interventions. We integrated qualitative interviews and focus group discussions with study participants to assess the acceptability of the treatments. We report on findings assessing the acceptability of treatment, and perceived benefits and/or challenges.

## Methods

### Study setting

The study was conducted in Kisumu County in western Kenya- a region where our team has established research collaborations for over ten years. The region has a high burden of illnesses as compared to other regions in Kenya, including high rates of mental health disorders and co-morbid conditions including HIV [56, 57].

### Study design and participants

#### About the parent trial

SMART DAPPER collaborates with Kisumu County Referral Hospital (KCRH), and its large, public sector, primary care outpatient clinics (~10,000 participants/month). The parent trial recruited outpatient adult participants with current Major Depressive Episode (MDE) and/or PTSD from public health facilities that cater to both primary and specialized care settings. The published study protocol provides additional details about the trial [20], while the details of the qualitative sub-study are described below.

#### Qualitative sub-study

Embedded within the parent trial, the qualitative study sought to document participant experiences with the intervention and interactions with the study team.

#### Study team

The qualitative study team comprised of six research assistants (5 female and 1 male) and two study coordinators (GR and AA) trained in qualitative data collection methods; they conducted and documented the in-depth interview (IDIs) and focus group discussions (FGDs). All were degree holders with training in the interview guides, qualitative data collection, and human subjects’ research. The analytical team included the lead author (MG), qualitative research consultant (IM), GR and AA (study coordinators), and study manager (RB); all were trained in qualitative research methods and had prior experience conducting, analyzing, and writing qualitative research papers. IM, GR, and JW are Kenyan female researchers, and AA a Kenyan-male researcher- all are from the communities where the research is conducted. MG is a US-based east African female researcher and RB is a US-based female researcher.

*Participants and sampling*. Eligible study participants were 18 years or older, primary care participants at KCRH who met the threshold for MDE and/or PTSD on the Mini International Neuropsychiatric Interview (MINI 7.0.2) and new to the study and team. We excluded and referred those who were identified as having a moderate or high risk of suicide, current/previous hypomania or mania, hazardous drug or alcohol use, severe cognitive dysfunction, were pregnant/breastfeeding, lacked capacity to give consent, unable to attend weekly treatment appointments or were receiving outside mental health care. The qualitative study participants were purposively sampled following their parent trial enrollment, balancing for sex (~15% male and ~85% female to reflect the parent study population), intervention arm (IPT/FLX), and for those who completed their treatment, no more than 3 months since their completion. We also sampled those who dropped out of treatment.

### Data collection

Six research assistants and two study coordinators (GR and AA) conducted in-depth interviews (n = 17) and three (n = 3) focus groups with an average of 4 participants (a total of n = 12 across all FGDs), between May 2021 to October 2021, in-person and utilizing semi-structured open-ended guides informed by an implementation science framework [20]. IDIs were conducted between May-September 2021, while FGDs were conducted between May–October 2021. All participants who were approached for the qualitative data collection agreed to participate. The IDIs and FGDs, combined, were adequate to achieve theoretical saturation. Emerging data and interview debriefs were conducted to determine if additional interviews were needed, at various stages of the data collection period. IDIs were conducted to delve into individual experiences with the SMART DAPPER intervention, while FGDs sought to explore group dynamics in discussing the various aspects of the intervention and mental health. The guides focused on the following areas: (1) conditions prior to the intervention start and general impressions about the intervention, (2) specific treatment experiences, (3) positive, negative, and neutral impacts of the intervention, and (4) interactions with the intervention and study team, including dimensions of respect, confidentiality, and care provision. IDIs and FGDs were conducted in the preferred languages of participants (Dholuo [LUO], Swahili, or English). IDIs were conducted one-on-one, while FGDs were conducted with 3–5 people in each group, at the health facility with only the participants and research team members present; FGDs lasted 1.5–2 hrs., while IDIs lasted 45 mins to 1.5 hrs. Audio recordings were directly transcribed and translated into English transcripts. Study team members reviewed transcripts and audio recordings to ensure the accurate capture of data.

### Data analysis

Our analytical approach was guided by constructivist grounded theoretical approaches and content analysis [58, 59]. The initial coding framework was refined at multiple stages. RB, GR, AA, and SM, along with research consultant IM, were each assigned three transcripts to review and summarize. They then met to compare notes and develop an initial coding framework based on review notes and discussions. IM and GR then applied the coding framework to the transcripts utilizing Dedoose software, discussing emerging themes and iteratively refining the coding framework; inductive codes were added at this stage and discrepancies resolved. The final coding framework included 12 parent codes and 23 child codes. The lead author (MG) queried all codes relating to the intervention impact, patient experiences, barriers, and facilitators. Based on a close reading of the coded excerpts, as well as an in-depth review of all transcripts, MG developed analytical summaries using MS Excel. This summary was reviewed and discussed with GR, AA, RB, and SM for consensus. We developed an analytical memo utilizing the socioecological framework (SE) [60] and health belief model (HBM) to present findings, including perceived severity (HBM) to describe the reasons for starting the intervention and the onset of depression/PTSD symptoms. We then present the impacts of the intervention utilizing the SE framework, which presents the perceived impacts of the SMART DAPPER intervention at the individual, interpersonal, and community and health systems levels.

### Participant compensation

The study provided travel reimbursement for participation in face-to-face study research data collection (not treatment) at 350ksh (~$3) per visit. IDIs and FGDs participants were compensated at this rate.

### Ethical approvals

The study was approved by the University of California San Francisco (UCSF) Human Research Protection Program Institutional Review Board (IRB), the Kenyatta National Hospital- and University of Nairobi Ethics and Research Committee (KNH-UoN ERC) and the Kenyan Pharmacy and Poisons Board (PPB), the National Commission for Science, Technology & Innovation (NACOSTI). All SMART DAPPER participants provided written consent to participate.

## Results

Among the parent trial cohort, the average age was 34 (ranging from 18 to 85); a majority were female (90.6%) and had at least some primary education (51.7%). At baseline, the majority of participants were diagnosed with major depression (93.2%) while nearly half (46.5%) were diagnosed with both major depression and PTSD. Only 1% reported a history of prior access to mental health care services. Notably, a large proportion (39.4%) of the study participants were living with HIV (Table 1). The qualitative study cohort was similar in overall characteristics. Individual-level participant characteristics are shown in Table 2.

**Table 1:** Baseline characteristics of the main study and qualitative cohort.

	IPT (n = 1082)	Fluoxetine (n = 1080)	Total (n = 2162)	*p* Value^1^	*Qualitative Cohort (n = 29)*
**Age**					
Age in Years (Median [min-max])	34 (18–85)	33 (18–77)	34 (18–85)		42 (28–70)
**Sex**					
Female	977 (90·3%)	981 (90·8%)	1958 (90·6%)	0·67	24 (82.8%)
Male	105 (9·7%)	99 (9·2%)	204 (9·4%)		5 (17.2%)
**Formal Education**					
None	16 (1·5%)	19 (1·8%)	35 (1·6%)	0·49	n/a
Some primary/primary	576 (53·2%)	541 (50·1%)	1117 (51·7%)		16 (55.2%)
Some secondary/secondary	389 (36·0%)	418 (38·7%)	807 (37·3%)		11 (37.9%)
Some college/ Certificate/ Diploma	101 (9·3%)	102 (9·4%)	203 (9·4%)		2 (6.9%)
**Baseline Diagnosis(es)**					
Major Depression	1009 (93·3%)	1006 (93·1%)	2015 (93·2%)	0·92	26 (89.7%)
PTSD	555 (51·3%)	563 (52·1%)	1118 (51·7%)	0·70	13 (44.8%)
Major Depression and PTSD	498 (46·0%)	508 (47·0%)	1006 (46·5%)	0·64	11 (37.9%)
History Mental Health Care	10 (0·9%)	13 (1·2%)	23(1·1%)	0·53	
**Co-morbidities**					
HIV	438 (40·5%)	413 (38·2%)	851 (39·4%)	0·29	17 (58.6%)
Other	99 (9·1%)	96 (8·9%)	195 (9·0%)	0·83	5 (17.2%)

^1^Chi-square test (*not reported for the qualitative cohort*)

**Table 2:** Baseline characteristics of qualitative study participants.

ID	Type	Age	Sex	Education	Marital Status	Diagnosis	HIV	First Line Tx Group	Second Line Tx Group	First Line Tx Status	Second Line Tx Status
3016	IDI	52	F	Some secondary/secondary	M	MDE and PTSD	N	IPT	NE	C	NA
3022	IDI	26	F	Some secondary/secondary	SP	MDE and PTSD	N	FLX	NE	C	NA
3023	IDI	35	F	Some college/Certificate/Diploma/Degree/Post-graduate	M	MDE	N	IPT	FLX + IPT	C	C
3044	IDI	35	F	Some primary/primary	SP	PTSD	N	FLX	NE	C	NA
3060	IDI	36	F	Some primary/primary	M	MDE	Y	FLX	NE	C	NA
3218	FGD	38	F	Some primary/primary	M	MDE	Y	IPT	NE	S	NA
3276	IDI	28	M	Some secondary/secondary	SP	MDE	Y	FLX	FLX + IPT	S	C
3318	FGD	45	F	Some secondary/secondary	M	MDE and PTSD	Y	IPT	NE	C	NA
3372	FGD	60	F	Some primary/primary	W	MDE and PTSD	Y	IPT	NE	C	NA
3444	FGD	42	F	Some secondary/secondary	M	MDE and PTSD	Y	IPT	NE	C	NA
3578	IDI	41	F	Some primary/primary	SP	MDE	Y	IPT	FLX + IPT	C	C
3633	IDI	58	M	Some secondary/secondary	M	MDE and PTSD	Y	IPT	NE	C	NA
3870	IDI	50	M	Some secondary/secondary	M	MDE	Y	IPT	NE	C	NA
4054	IDI	30	F	Some primary/primary	NM	MDE	N	FLX	NE	C	NA
4212	IDI	20	M	Some college/Certificate/Diploma/Degree/Post-graduate	NM	MDE	Y	FLX	NE	C	NA
4329	IDI	46	M	Some secondary/secondary	M	MDE and PTSD	N	IPT	FLX + IPT	C	C
4493	IDI	44	F	Some primary/primary	SP	MDE	Y	IPT	NE	C	NA
4540	FGD	40	F	Some primary/primary	M	MDE	Y	FLX	NE	C	NA
4613	FGD	54	F	Some primary/primary	W	PTSD	Y	FLX	NE	S	NA
4657	FGD	44	F	Some secondary/secondary	W	MDE and PTSD	Y	FLX	NE	S	NA
4713	FGD	46	F	Some secondary/secondary	M	MDE	N	IPT	NE	S	NA
4719	IDI	31	F	Some primary/primary	M	MDE	N	FLX	NE	S	NA
4801	FGD	32	F	Some secondary/secondary	M	MDE	N	FLX	NE	S	NA
4814	FGD	46	F	Some primary/primary	M	MDE	N	IPT	NE	C	NA
5111	FGD	48	F	Some primary/primary	M	MDE and PTSD	Y	IPT	NE	C	NA
5246	FGD	70	F	Some primary/primary	SP	MDE	N	FLX	NE	D	NA
5265	IDI	38	F	Some primary/primary	SP	MDE and PTSD	Y	FLX	NE	D	NA
5443	IDI	41	F	Some primary/primary	W	MDE	Y	IPT	FLX + IPT	C	C
6585	IDI	47	F	Some primary/primary	M	MDE and PTSD	N	IPT	NE	C	NA

Sex- M-male F-female, Marital status: M-married, NM-never married, SP-separated, W-widowed; MDE- Major Depressive Episode, PTSD-post traumatic stress disorder; HIV status: Y- yes, N-no; Tx (treatment) groups and assignment status- C- completed Tx, S- started Tx, D- declined Tx

### Qualitative findings

We present our findings utilizing aspects of the health belief model: perceived severity as reasons for starting the intervention and discussions of the onset of depression or PTSD symptoms. We then present the impacts of the intervention utilizing the SE framework, including at the individual, interpersonal, and community and health systems levels; the majority of the findings were at the individual and interpersonal levels. We then discuss the cues to action (i.e. the support and facilitators towards the intervention), as well as perceived barriers (challenges towards the intervention).

### Perceived severity

Participants discussed the perceived severity of their conditions including detailed accounts of their symptoms as well as the varied circumstances they perceived caused stress including family and marital conflict, loss of a child, loss of income or a job, and a traumatic event such as a death or illness.

Some participants cited family-related conflict:

*“I had a problem of land, with my brother and stepbrothers; we have two mothers and the other house. So, it was a serious one [the fight]. All these things- school fees is there, the children want to go back to school, some are going to college, there was no money, and I was really depressed.”* ***3633, 58, male***, ***IPT, completed, English***

Others attributed their symptoms and described the severity of their marital and relationship challenges, including the loss of a child:

*“At that particular time, I was having some psychological problems. Such as, at that time I lost my marriage, broke up with my wife, she ran out and somehow again, I came and married another wife. And with her [new wife], I came to lose my child [child died]. So, in that duration, I met the SMART DAPPER [team] members. I was having so much stress in life.”* ***3276, 28, male***, ***2nd line combination, completed, English***

Some recounted the severity of their condition, including their marital problems which had arisen to a crisis level where they had considered suicide:

*“When I came [to SMART DAPPER], I had a problem with my marriage […] it reached to a level of committing suicide but am so grateful to SMART DAPPER for the counseling they gave me. I started getting treatment. I appreciate it so much, because I changed.”* ***3016, 52, female***, ***IPT, completed***, ***Kiswahili***

For others, economic insecurity and income loss were particularly salient:

*“Another important challenge that I have is that, at my age, I don’t have my own home. I am still living in a rental house, where I also have rent arrears of eleven thousand and something that I need to give the landlord. Whenever I receive her [landlord’s] call, I get stressed again. It happens because I don’t have money and I don’t know what to say or tell her.”* ***3578, 41, female, 2nd line combination, completed, Luo***

Others were stressed about job losses, including that of their family members, which was also reported to lead to marital problems:

*“The business was doing so badly, yet there are loans that I was paying off. So, I had a lot of pressure from the lenders because they kept on calling me. Yet, there was nothing coming out of the business. I was just depressed. I was wondering, ‘where will I get money to pay these people?’ because they will put me to shame, they will come and start repossessing things.”* ***3023, 26, female, 2nd line, combination, completed, Luo***

For others, a traumatic event or a family member’s illness or death was reported to instigate their PTSD:

*“It is because of the life I had at that moment. It was a bad life. At that time, I was going through a lot. My mum was in a hospital- she was in a coma and then she died.”* ***4329, 46, male, fluoxetine, completed, Luo***

### Impacts of the intervention at the individual, interpersonal and community and health systems levels

#### Individual level impacts of the intervention

We use the SE framework to discuss the individual impact of the intervention across somatic and psychological health domains. These include positive changes such as a reduced headache, improved appetite and weight management, increased energy, improved sleep, and better self-efficacy.

Participants reported a reduction in headaches, as compared to their experience prior to the intervention.

*“I noticed a difference because, as it was getting to the point where I felt like the headaches subsided. My heart also stopped beating as it used to, as I continued with the treatment. I even started to gain strength.”* ***4054, 30, female, fluoxetine, completed, Luo***

Similarly, participants also noted a change in their heartbeats, in addition to reduced headaches.

*“I just encouraged myself to take the drugs and hope for a better result as times goes by. The drugs worked so well, and the constant headaches started reducing; my fast heartbeat also stopped.”* ***3060, 36, female, fluoxetine, completed, Kiswahili***

Others experienced positive impacts on their appetite and weight management. For some, it meant the realization of weight loss goals, while for others, the intervention facilitated weight gain and attainment of a more ideal body size.

*"So, at that point, I was 64kgs. And I believe when you’re sad, you don’t want to eat. You don’t have an appetite, so I used not to eat. Like, I used to skip some meals not because they are not there, but I didn’t have the desire to eat. So then after the treatment, I started to develop an appetite, I started eating a lot and currently, I am 75kgs. That’s a gain of 11kgs.”* ***3060, 36, female, fluoxetine, completed, Kiswahili***

This regulated appetite meant increased energy and ability to take on physical activities:

*"First, my health was deteriorating, and I didn’t even have energy- eating was a problem. But for now, I appreciate it so much that I have good health, I am taking care of myself; I have tried so hard to lose weight and now I have good health. I can now walk a long distance, even from here to XXX, without any problem.”* ***3016, 52, female, IPT, completed, Kiswahili***

Many participants also reported improved sleep as a positive impact of the intervention, which also led to better functioning in day-to-day activities.

*"IPT treatment has improved my life for the better. I didn’t have many friends before, but now I have many friends. I slept well today; initially, I couldn’t sleep even sleeping hours. Working becomes hard because I would feel sleepy during working hours. Currently I sleep well, I do my job well and get home with some cash to support myself. I received much support from SMART DAPPER."* ***FGD3, P4, IPT, Luo***

*"There is a difference*, *before and after*, *because during that time my heart was aching*. *I could not sleep; I could wake up and just sit down*. *But now I am sleeping*, *even during the day*, *I can sleep*.*”* ***3023*, *35*, *female*, *2nd line combination*, *completed*, *Luo***

Participants also noted the important benefits of the intervention including improved self-efficacy and ability to manage challenges:

*“That I saw that it wasn’t bad or good after I had finished, I understood that I am the one with the problem, so I am the one to decide how I will live. Because they [SMART DAPPER] have really helped me.”* ***3016, 52, female, IPT, completed, Kiswahili***

*“The treatment that I received changed me and as of now*, *even if I experience any stress*, *I know how I can handle it […] if I experience any challenge*, *I know how I can handle it*.*”* ***4493*, *female*, *IPT*, *completed*, *Luo***

For others, this improved self-efficacy reportedly led to better self-worth:

*“I can say that treatment changed my emotions and life because I used to feel like I was a useless person by asking myself too many questions. But treatment enabled me to know myself better…”* ***3060, fluoxetine, completed, Kiswahili***

Some participants reported acceptance of and normalized reactions to everyday stressors, including their perceived control over some aspects of their lives:

*“After being counselled, I was told that there are some things that we can’t control as individuals. So, I also decided to embrace that and accept my future. I also decided to embrace that because the economy was also worsening, there was nowhere to get money from, therefore, it was upon me to work harder […] before treatment, I was easily irritated. Sometimes I could be lonely and just sit by myself and if anyone upsets me, I would really be mad; but now things are just so normal to me. I am not concerned with a lot of things like I used to be. Things are just normal to me."* ***3023, 35, female, 2nd line combination, completed, Luo***

Other participants reported increased concentration, which for some, meant increased productivity:

*"Today, my memory is good. I can perform many functions soberly as a human being. I remember immediately, any pending duties, then I make sure I complete it."* ***4719, 31, female, fluoxetine, dropout, Luo***

Participants also reported improved cognitive functions, describing a resumption of routine functions:

*"When they see me now, unlike before, I can do a lot of things by just thinking about them alone without asking somebody, which means I am mentally good. Everything in my brain is thinking quickly, as compared to before. I am normal […] you know when you are stressed, you are not even normal. So, it has helped me a lot. As at now I am so active, I can do most of my things, I can think positively all the time, I can do everything I wanted.”* ***3022, 26, female, fluoxetine, completed, English***

Improved health was reported to result in increased economic prospects and capacities. For some, improved social relationships, opened doors to jobs or other opportunities:

*“After getting treatment, my economic status has improved a lot, and this is happening because of the friends I have made. Nowadays, I cannot sit at home because I get other work through friends, which is changing my life."* ***3870, 50, male, IPT, completed, Luo***

For others, the positive change and increased energy brought on by the intervention made it possible to resume their daily chores and livelihood activities:

*“You know, at first, I wake up and just sit. You don’t feel like doing anything in your house. You don’t feel like doing anything, you just feel tired the whole day. But as I was engaged in this treatment, I’m always very strong. I can do my jobs very early. I even wake up at 4:00am and do everything, wash everything in my house, and go sell these vegetables. When I come back, I can even wash during the night. So as of now, my energy has increased a lot. I am so energetic.”* ***3022, 26, female, fluoxetine, completed, English***

Participants also reported better concentration, and improved cognitive functions, which they reported their ability to complete their duties:

*Before I could not concentrate, I used to lose memory, and my thinking was anticlockwise. But when I started on medication, all the depression and stress ended. Now I can concentrate and do my business as usual […] it didn’t end at once, but step-by-step, as I was attending and adhering to my treatment visits. The more I was taking my medication, the less stress and depression, and finally I was cured fully. I can now concentrate and attend to my duties and responsibilities better.”* ***3044, 35, female, fluoxetine, completed, Luo***

Finally, some participants reported a decreased reliance on other medications to manage their symptoms, and thus, decreased expenses.

*"At first, I would buy drugs. I was always tired, I would go and buy this one, when I am feeling headache […] I used to spend a lot of money buying drugs. But when I started this treatment, I don’t know but I just got sick in July last year with malaria only. When I compared the money, I was using at first to now, I feel so good. I am not using a lot of money to go and buy so many drugs. This treatment has really helped my life.”* ***3022, 26, female, fluoxetine, completed, English***

### Interpersonal level impacts of the intervention

The most salient findings were those relating to positive changes in the relationships with partners, immediate family members, and others within the community. Participants credited the intervention for increasing their ability to stay calm and avoid unnecessary conflicts, increased harmony, better conflict resolution, and improved relationships with friends and family.

A patient reflected on their past quick irritability and conflict-prone approach, noting:

*“Just as I have told you earlier, during that time, if I was in the company of other people, I would easily get irritated. Whenever someone would say something, I would think that they never respected me because of this or that. But now I’m okay. Yes, I take things normally. Before, then, I would argue a lot and I would portray arrogance- but now, most of the time, I just keep calm. Yes, it is not very easy for me to have conflict with others.”* ***4329, 46, male, 2***^***nd***^ ***line combination, completed, Luo***

Others also reflected on their conflict avoidance, noting a recent shift to focus on reconciliation and harmony:

*“I never used to talk to someone if they were in any disagreement with me. In relation to what I learnt through the treatment provider [name], I just reconciled with the person for me to have a peaceful heart. Because before, I used not to do that; but now I am the person who will have the burden [to make peace], and that is helping in reducing stress. I can now go to the person with whom I have grudges and talk to them.”* ***4493, 44, female, IPT, completed, Luo***

Another patient, despite later dropping out of treatment, still describes how encouragement from her intervention therapist led her t resolve a conflict between her and her co-wife:

*" I noticed a change. I can even say that I told my therapist that because of stress, I don’t even have a friend. I am in the same home as my co-wife, but we don’t talk. This really caught the attention of my therapist, and she could ask me if there is any change. Indeed, after some time, I noticed some changes because we are only two of us in that home, but we don’t talk. But after that, I noticed some changes between us. I can say that because she used to ask me this again and again, ‘how is it between you and your co-wife?’ I decided that even if she [co-wife] will think I am stupid, I am just going to talk to her.”* ***5443, 41, female, IPT, drop out, Luo***

Others noted improvements in relationships with others outside of their family, including neighbors:

*“I like coming [to IPT] because I could heal without taking medication. I don’t adhere well to medication, so I loved IPT so much. Secondly, since I got enrolled in study I had positive change in my life, I said initially that I was short tempered and feel isolated most of the time, but when I started IPT, I changed and become rejuvenated. Even my neighbor would realize that I have improved. Some of my neighbors would want to know which place I go to for support, but I told them I have a provider who supports me […] when I completed treatment, one of my neighbors also enrolled in SMART DAPPER and currently receiving treatment too…"* ***FGD3 P1, IPT, Luo***

For some participants, addressing their own depression and stigma meant they were able to encourage others:

*“Yes, when we were discussing, I was, initially, I was not very free to tell anybody about my status; we discussed and even after about the fourth week, in fact I manage to counsel about five people.”* ***3633, 58, male, IPT, completed, English***

### Cues to action

#### Facilitators of the intervention

Participants discussed components of the intervention they perceived to be important for their success. These were credited for motivation to remain engaged in the intervention.

Participants were modestly reimbursed transport costs to attend in-person research assessment visits with blinded clinical evaluators (9 visits, total of ~$25 distributed over 2 ½ years). The study reimbursement for transport related for data collection was reported to support participants in buying food and alleviating food insecurity:

*“I can say that as a business lady, when I go to sell my goods, I can sit there without selling anything. When I come for treatment visits, I know that I will get three hundred to four hundred shillings, which is enough to buy maize flour. I will also get milk to make tea because I have not taken any food. That is all I can say, helped me a lot.”* ***4493, 44, female, IPT, completed, Luo***

*"Sometimes*, *because of the situation of COVID-19*, *there was no money and when I am called*, *I could recall that sometimes they [SMART DAPPER] give Ksh*. *350*. *That was enough for me to eat*. *Sometimes*, *that was motivation*. *I could say that when I go there*, *I will get Ksh*. *350 therefore I will not sleep hungry because sometimes business was not good*.*"* ***3023*, *35*, *female*, *2nd line combination*, *completed*, *Luo***

Others perceived the warm and welcoming environment, particularly among providers, as a motivating factor:

*"What encouraged me most are the good treatment services which I received; I liked the type of counselling I was offered at SMART DAPPER. The treatment improved my life, and I could not miss a session. Secondly, my IPT provider was a very nice lady, she could remind me three days to my treatment visit through phone call, then a day before, she can call to confirm whether I will attend or not. So, my provider’s good attitude and connection was also encouraging me to honor my treatment visits."* ***FGD3- P2, IPT, Luo***

*"What really motivated me was the level of love all SMART DAPPER staff were showing towards participants*. *You’re given warm welcome and assisted to find your provider […] you would feel happy*.*"* ***FGD3 P1*, *IPT*, *Luo***

A patient notes the flexible and ideal scheduling of IPT sessions as a motivator:

*"There were no negative aspects because during treatment, everything was done in a very organized way, and it did not interfere with my schedule. I also had enough time to plan for the appointment date without any challenges, which made my work easier too […] I was able to attend all the treatment sessions in person without missing even one session.”* ***3870, 50, male, IPT, completed, Luo***

This was particularly important when participants felt they were late for their appointments:

*"The way they treated me with respect is that there was privacy, at least when you arrived, they take you direct to the room […] then the way they talk to you also. They will not treat you harshly even when you are late maybe for the clinic those…even if you are absent or late but you communicated at least they get to know what happened […] they take you the way you are, they understand why you are late and the reasons, and what can be done so that next time, you don’t come late."* ***4212, 20, male, fluoxetine, completed, English***

Further, participants appreciated the confidentiality provided during intervention participation:

*"My treatment provider X would assure me of confidentiality before we start. The provider assures me to keep every talk very private and confidential, unless there is talk of committing suicide, then she may refer to you to another senior provider who will also keep your information confidential […] my provider showed me a lot of respect and I shared all my problems with her until my life improved […] I thank her so much."* ***FGD3, P4, IPT, Luo***

For some, the study participation and intervention impacts were so profound that they encouraged others to participate, noting subsequent positive changes among their friends:

*" It has changed my life […] it has helped me with my friend. There at the estate, I also picked some of my friends who also had that problem [mental health] and brought them here. I am seeing their lives have changed. Because when they came here, the doctors talked to them, they were counselled, and now I am seeing they are good."* ***3022, 26, female, fluoxetine, completed, English***

#### Barriers to intervention engagement

Participants noted some challenges that impacted their participation in the intervention. Some participants reported livelihood and scheduling-related conflicts:

*“the only negative experience I had was about time, sometimes I could have limited time to come for sessions at KCRH-Smart DAPPER then I could find my IPT provider X still having session with another participant, I will have to wait for her to finish, by then customers are also waiting for me at my business premise so that’s the only problem I had during my treatment period…”* ***FGD3 P2, IPT, Luo***

Others recounted the stigma associated with the physical space where the intervention is implemented, as well as having mental health issues, noting:

*"Let us say you are in PSC (patient support center), you can know what the other person is thinking. Like number three had said, he thought they treated mentally challenged people. Another person who has the same mentality will conclude that the people there are mentally challenged."* ***FGD 4 A2, fluoxetine, Luo***

**“***Because let’s say for example*, *your friend is passing by and he sees you seated at the waiting bay [in clinic]*, *and it is known that the study is for people with mental health*, *depression and anxiety*. *When he goes out there*, *he’ll start saying; ‘you see so and so has mental problem’*. *You see that creates stigma and more depression*. *So that’s why sometimes I opted to coming in the evening […] the waiting area was kind of uncomfortable […]You could take like 1–2 min as they searched for an empty room*. *So at least*, *that was a plus*.*"* ***4212*, *20*, *male*, *fluoxetine*, *completed*, *English***

Some participants also noted major life events or crises that impacted their engagement:

*" The challenge that I faced was when I was admitted inpatient. During this time, I was delaying taking medication and the provider would quarrel with me and urge me to let them know when I’m admitted. At some point, I was admitted and forgot to carry my pills."* ***4719, 31, female, fluoxetine, dropout, Luo***

*“[I had an] accident whereby my house burned*, *and I also lost a child*. *That was the thing that made me disappear- not to come back for another re-fill again*. *So*, *I got around three months away*. *But they [study] used to contact me*, *that’s the best thing that they used to do*. *They used to contact me and confirm if I’m there or not*. *I used to tell him that I’m there but right now I’m [not in town]*.*”* ***3276*, *28*, *male***, ***2nd line combination*, *completed*, *Luo***

A patient also discussed their preference for the use of traditional medicines rather than the prescription study drugs:

*"The session [IPT] was good. I am the one who said I don’t take medication and I also don’t like injections; I just use traditional medicine. I like using traditional medicine compared to going to the hospital […] I told them that I cannot take the medication, yet I am not going to use them. Taking the medication yet I am not going to use them will not give me peace.”* ***FGD 5 P3, dropout, Luo***

Finally, some participants found difficulty with the study enrollment counseling, the randomization procedures, and subsequent assignment to a treatment arm:

*I fell on the Fluoxetine arm and that is where my problems started. I wanted to get a better understanding before the initiation, but the treatment provider was not willing to do so. Not that I refused to take the drugs, even though they are drugs for stress, but I haven’t used it before. I didn’t know the effects of that drug […] I expected to be explained whether the drugs can make someone weak, have headache or diarrhea and the response was ‘you are supposed to ask me the questions after you have started the medication’, I became tired."* ***5265, 38, female, fluoxetine, drop out, Luo***

*“I asked questions then the procedure was like*, *I met a psychologist and we talked*, *she elaborated*, *she told me like it’s a study […] they developed a software and it’s a study*, *so it either is ‘you will be taking the drugs*, *or you will [get therapy]’*. *The category you will fall into*, *the machine is what will determine*, *so I was not comfortable*.*”* ***6585*, *47*, *female*, *IPT*, *dropout*, *English***

## Discussion

The study explored user experiences with the SMART DAPPER intervention; narratives about the severity and onset of depression and PTSD recount family and martial conflict, loss of a child, loss of income or a job, and a traumatic event such as a death or illness. We found that impacts of SMART DAPPER reported at the individual, interpersonal and community levels. At the individual level, findings reveal positive somatic and psychological health outcomes including reduced headaches, improved appetite and weight management, and improved sleep. Participants noted increased self-efficacy, mood regulation, improved cognitive functions, and better concentration, which were reported to lead to increased economic opportunities. At the interpersonal level, participants reported an overall reduction in conflict, better conflict management and resolution, increased harmony with family and community members, and improved relationships with their partners and children. Others also discussed perceived challenges included balancing the intervention with livelihoods, preference for traditional medicines, actual or anticipated side effects with medication (FLX), mental health stigma, major life events, as well as perceived inadequate counseling and challenges with providers.

Our participant narratives reveal the severity of events that precipitated depression and/or PTSD including economic insecurity, job loss, marital and family conflict, and loss of family, as previously documented [28, 61–64]. Marital conflict, lack of partner support, and marital dissolution were all reported to contribute to depression and PTSD, suggesting that relationship conflicts have profound implications for mental health, as demonstrated in similar settings [28, 64–66]. Further, in SSA, a region where an estimated 67% of PLHIV reside [16], rates of depression and post-traumatic stress disorder (PTSD) are elevated. A large proportion of our study cohort (~40%) were living with HIV, also underscoring the unique challenges of managing HIV alongside depressive and stress-related disorders, as has been extensively documented by others [11–15].

Impacts of SMART DAPPER were reported at the individual, interpersonal and community levels. At the individual level, participants noted somatic and psychological improvements in their symptoms of depression and PTSD. These gains included increased energy, improved sleep, and fewer headaches. Further, others reported improved mood, cognitive function, and concentration. Concurrent findings have previously been reported in similar settings [66, 67]. For example, a study in Uganda demonstrated an improvement in work status and increased functioning following depression treatment [68]. Many of the reported positive impacts also spilled over to the interpersonal level; participants reported reductions in conflict, better conflict management and resolution, increased harmony with family and community members, as well as improved relationships with their partners and children, as previously documented [27, 66, 69]. A recent study of Kenyan community health workers (CHWs) delivering IPT among adolescents demonstrated how youth were better able to effectively communicate, interact with others, manage their anger, and resume work, also reported by participants in our study [70]. Similarly, adolescents in Kenya demonstrated increased confidence in speaking up about problems and expanded social networks, following treatment [71]. Further, our participants, consistent with studies in Ethiopia, South Africa, and Uganda, describe increased social networking opportunities following therapy [25, 28, 72]. Finally, the respect, confidentiality, and transport reimbursement (for assessment, not treatment) offered during study were perceived to facilitate continued engagement. The transport reimbursement offered in our study was reported to offset economic hardship, which is a documented barrier to services [73]. The tele medicine aspect of SMART DAPPER, i.e., IPT sessions offered via phone, may have alleviated this burden. However, it remains a challenge that must be considered when scaling other programs and interventions. Participants in our study also recommended the program to others, having realized the personal benefits, similar to participants in a recent study of group IPT in Senegal [73].

However, we also noted several challenges. Among them, participants noted that the stigma associated with mental health services and physical location of the SMART DAPPER facilities. This has been documented by others who have underscored challenges related to patient confidentiality and privacy, as well as the stigma associated with mental health service provision, especially in the context of HIV [73, 74]. Further, participants noted conflict with livelihoods, preference for traditional medicine, actual or anticipated side effects with medication, mental health stigma, major life events, as well as inadequate counseling and challenges with providers. Previous studies, including a recent systematic review, have documented similar attitudinal and economic barriers to mental health services including stigma or anticipated stigma, discrimination, mental health literacy, as well as societal beliefs in alternative treatments [28, 73–76].

Our findings are particularly important in SSA where many patients face challenges in accessing treatment due to many challenges at the individual, health systems and community levels [5–10, 77–79], underscoring the importance of non-specialist delivery of combination treatments such as SMART DAPPER. Our study contributes important qualitative insights about experience with interventions to address the burden of mental health illness in the region; it adds more data to the efforts to expand mental health access including increased training, capacity building, and task shifting of mental health services [19–23]. Our qualitative findings, and the reported perceived benefits show that Interpersonal therapy (IPT) can address mental health challenges. This adds to previous literature showing the utility of IPT in addressing complex social cultural and personal dynamics which contribute to the burden of mental health illness and health outcomes [20, 21, 24–29]. Further, SMART DAPPER may contribute to similar studies non-specialist delivered IPT, including in the context of HIV care [21–25, 29, 30]. Finally, we demonstrate that fluoxetine (FLX), widely used in other high-income settings and a key pillar of SMART DAPPER, can be utilized to manage depression and stress-disorders, concurrent with previous findings [20, 80, 81]. Our study contributes critical information on the potential impact of combination therapy (IPT and FLX) and provide data on the user acceptability for the management of mood and stress-disorders.

### Policy implications

The parent trial also convened an Implementation Resource Team (IRT), a group of key stakeholders who were identified to facilitate the implementation and scale-up of the study findings. The IRT includes clinic staff, patients and providers, local health policy and community leaders, regional stakeholders, and national mental health policy experts as stakeholders; our findings, along with the input and guidance from the IRT, will be key to addressing the challenge of translating evidence-based practices into broader practice and policy. Our qualitative study directly informs policy at the local and national level, including providing critical data on patient acceptability of this treatment implementation strategy as well as barriers that must be overcome to inform the scale up of similar interventions in Kenya and SSA, broadly.

## Strengths and limitations

We note several limitations in the study. First, the interview and focus group guides were developed utilizing an implementation science (ImS) framework and thus may not have delved deeply into personal motivations, opportunities, and capabilities of participants. Data were limited to implementation-related domains, including the perceived impact of the intervention. However, we drew on the health belief model (HBM) to explore data to demonstrate these themes (perceived severity). Second, as subjects who were lost to follow up (LTFU) are inherently more difficult to engage, they may not be well-represented in the narratives. However, we note that the number of participants that terminated from the study was very low (6.8%). Third, participants may have been more likely to give favorable feedback due to social desirability bias. However, we implemented several strategies to mitigate these limitations. Participants were interviewed by study staff who were not implementing the intervention and were encouraged to provide their honest feedback. We utilized several differing strategies including conducting both IDIs and FGDs with participants who dropped out of treatment to include the diverse perspectives of those who did and did not successfully complete the intervention. Finally, our analysis and interpretation of findings utilized a group-based approach inclusive of those who collected the data, which strengthens the rigor of our findings.

## Conclusions

To our knowledge, SMART DAPPER is the first study in SSA to provide combination psychotherapy and pharmacotherapy for the treatment of major depression and PTSD among participants in Kenya; we documented critical patient experiences with the intervention to inform future scale-up. Data demonstrate the promise of the interventions and highlight the barriers, challenges, and facilitators that must be addressed when scaling similar interventions. Our study contributes critical and timely information to guide the implementation and integration of mental health services using local non-specialists.

## Supporting information

S1 Checklist Inclusivity in global research SMART DAPPER. (PDF)

S2 Checklist COREQ checklist. (PDF)

S1 Text SMART DAPPER study protocol. (PDF)

S2 Text SMART DAPPER IDI and FGD guides. (PDF)
