# Supplementary material for: “The peace that I wanted, I got”: Qualitative insights from patient experiences of SMART DAPPER interventions for major depression and traumatic stress disorders in Kenya
Source: PLOS Glob Public Health. 2024 Sep 5;4(9):e0002685. doi: 10.1371/journal.pgph.0002685 (PMC11376547; doi:10.1371/journal.pgph.0002685)
Supplement: S1 Checklist — (PDF) [file pgph.0002685.s001.pdf]

# Inclusivity in global research

PLOS' policy on inclusivity in global research aims to improve transparency in the reporting of research performed outside of researchers' own country or community and ensures that PLOS publications reporting global research adhere to high standards for research ethics and authorship. Authors of relevant research articles may be asked to complete the questionnaire below, which outlines ethical, cultural, and scientific considerations specific to inclusivity in global research. This questionnaire may be requested when researchers have travelled to a different country to conduct research, if research uses samples collected in another country, research with Indigenous populations or their lands, or if research is on cultural artefacts. Researchers travelling to another country solely to use laboratory equipment will not normally be required to complete the questionnaire. However, the questionnaire can be requested at the journal's discretion for any submission – if you have been requested to complete this questionnaire by the PLOS journal you submitted to, please do so.

Please complete the questionnaire below and include this as a Supporting Information file with your manuscript. Note that if your paper is accepted for publication, this checklist will be published with your article in the supporting information files. Please ensure that you reference the checklist in the main body of your manuscript. We suggest adding a subsection 'Inclusivity in global research' to your Methods section and adding the following sentence: "Additional information regarding the ethical, cultural, and scientific considerations specific to inclusivity in global research is included in the Supporting Information (SX Checklist)"

The questions have been designed to be applicable to a wide range of study types, and there are subsections for both human subjects research and non-human subjects research. If any of the questions are not relevant to your research please mark them as "N/A" as appropriate.

## Ethical considerations, permits and authorship

*This section is applicable to all research types.*

Provide details as to who granted permissions and/or consent for the study to take place in the Methods section of your manuscript. This should include the names of **all** ethics boards, governmental organizations, community leaders or other bodies that provided approval for the study. If individuals provided approval refer to these people by their role or title but do not list their name(s).

| |
|---|
| Reported on line number: 215-220 |

If there were any deviations from the study protocol after approval was obtained please provide details of these changes in the Methods section of your manuscript.

| |
|---|
| Reported on page number: N/A |

Did this study involve local collaborators that are residents of the country where the research was conducted or members of the community studied? If you do not have any authors from said communities, please provide an explanation for this below.

Yes- our authorship team is inclusive of many collaborators that are residents of the country where the research was conducted or members of the community studied. Our data analysis process also was inclusive of collaborators who collected the data and could help interpret the information. See lines 152-162.

Everyone listed as an author should meet PLOS' criteria for authorship and all individuals who meet these criteria should be included in the author byline, rather than the acknowledgements. For further information please see the journal's Authorship Policy.

## Human subjects research (e.g. health research, medical research, cross-cultural psychology)

Did you obtain written informed consent from a representative of the local community or region before the research took place? How did you establish who speaks for the community? Details of written informed consent obtained from study participants should be reported separately in the Methods section of your manuscript.

Yes- the study obtained approvals at the national, regional (County) and local (hospital) levels as is required for most research implemented in hospital settings. The study also obtained approval from the National Commission for Science, Technology, & Innovation.

How did members of the local community provide input on the aims of the research investigation, its methodology, and its anticipated outcome(s)?

The Implementation Resource Team (IRT) is a group of key stakeholders who were identified to facilitate the implementation and scale-up of the study findings. The IRT included clinic staff, patients and providers, local health policy and community leaders, regional stakeholders and national mental health policy experts as stakeholders.IRT members are involved from the beginning of the study to:

1. Address challenges of translating evidenced-based practices into the broader application
2. Adapt the interventions to the local context to increase the likelihood of sustainability and scale-up
3. Reduce the lag between development and testing of evidence-based practices and implementation
4. Prioritize variables likely to play key roles in implementation efforts
5. Foster partnerships between the study and local community
6. Share findings with the community as they become available

When engaging with the local community, how did you ensure that the informed consent documents and other materials could be understood by local stakeholders?

Throughout the consent process, the clinical evaluator ensured that the prospective participant understood each section in the consent before moving on by asking her or him to repeat it in his or her own words. Each point must be understood by the prospective participant, as indicated by their accurate repetition of each point in his/her own words. After they repeat the point accurately, the person obtaining the consent must initial after the point and only then may move on to the next point. If the prospective participant does not accurately repeat the information on the consent, it may be read to him/her two more times (for a total of 3 times). If, after 3 repetitions, the prospective participant cannot accurately restate the point, then they will deemed unable to consent to the study and therefore ineligible (see exclusion criteria). Prior to the signing the consent, the study staff should review each initial line to ensure that they have all been discussed and initialed.

Will the findings of the research be made available in an understandable format to stakeholders in the community where the study was conducted (e.g. via a presentation, summary report, copies of publications, etc.)? Please provide details of how this will be achieved.

The study team has annual Implementation Resource Team (IRT) meetings in Kisumu with a range of stakeholders. Study findings were presented at these meetings. Local media (television, radio, and print) have also published stories based on the study findings presented at the IRT meetings. The study findings were also disseminated to stakeholders in Nairobi, Kenya.

## Non-human subjects research using specimens/ animals collected as part of the study, or those housed in archival collections. Examples include archaeology, paleontology, botany and zoology.

Did the permission you obtained from a local authority to perform the study include an agreement on access to outputs and benefit sharing? This may include procedures to enable fair distribution of the benefits and resources arising from the research performed. Please include any details of Prior Informed Consent and Benefit Sharing Agreements obtained. These may be required by field-specific regulations, for example the Convention on Biological Diversity (CBD) and the associated Nagoya Protocol.

N/A

If the material used in your study was imported, please A) provide the year it was imported and B) indicate whether permits were obtained to import/export the materials used, C) provide details of any permits obtained. If this information is not available, please indicate this.

N/A

If you used archival specimens, please state how the material used in your study was acquired by the institute it is held in and provide details of any permits obtained for the original excavations/ sample collection. If this information is not available, please indicate this.

N/A

How was the potential cultural significance of the materials collected in your study to local communities considered in your research design? Were Indigenous peoples and/or local researchers and institutions involved with archaeological excavations / collection of specimens? If so, please provide a description of their involvement.

N/A

If your manuscript includes photographs of human remains please indicate whether authors obtained permission from descendants or affiliated cultural communities to do so.

N/A
