# Supplementary material for: “The peace that I wanted, I got”: Qualitative insights from patient experiences of SMART DAPPER interventions for major depression and traumatic stress disorders in Kenya
Source: PLOS Glob Public Health. 2024 Sep 5;4(9):e0002685. doi: 10.1371/journal.pgph.0002685 (PMC11376547; doi:10.1371/journal.pgph.0002685)
Supplement: S2 Text — (PDF) [file pgph.0002685.s004.pdf]

<u>Study Participant – IDI and FG Guide - Intervention</u>

INTRODUCTION

Thank you for speaking with me. We are talking to women and men who have been a part of SMART-DAPPER. Today I would like to hear more about your experience. I am interested in any recommendations/suggestions you have for the program. It's ok if you can't remember some details - I am more interested in your overall experience. There are no right or wrong answers to these questions and I will not be offended if you share your thoughts, both positive and negative. I am most interested in learning about what worked and what didn't work during experience with SMART-DAPPER. Your feedback will help make this and future programs better. I want you to speak most of the time and I will speak very little. Please remember that anything you tell me is confidential and will not affect your treatment or care that you receive in any way.

****GO THROUGH INFORMED CONSENT PROCESS****

Do you have any questions about this interview or the consent? Are you willing to be recorded? OK…let's go ahead and get started. Please stop me if you want to take a break, or if would like any part of interview to be unrecorded. Let's begin with some background questions about you.

Treatment experience

(1) I would like to hear how you first heard about SMART-DAPPER and your initial impressions

(2) How was your overall experience with the treatment that you received? What are the positive, negative and neutral aspects of the treatment?

(3) Were you able to attend all/most of the treatment sessions? Please tell me more.
   a. If so what helped you attend the sessions? If you could not attend all of them, why not?
   b. How was the frequency of the treatment visits?
   c. How was the length of the treatment session?

(4) Tell me more about your experience with the treatment provider.
   a. Were you able to ask questions during the sessions? If so, were the responses useful?
   b. How did the treatment provider treat you? Did you feel respected?
   c. Did you feel like your confidentiality and privacy were respected?
   d. Did you receive the attention that you needed?

e. Did you visit occur at the scheduled time?

(5) How was space where the treatment was provided?

a. Was it convenient? Clean? Comfortable? Private?

(6) Next, I'd like to hear how the treatment(s) changed, if at all, how you feel mentally?

a. Why were they helpful/non-helpful?
b. How, if at all, has your treatment affected your mood? Probe on reasons why/why not participation in treatment affected mood
c. How has treatment affected other aspects of your emotions?
d. How has it affected your concentration and attention?
e. How has it affected your energy?
f. How has it affected your appetite and weight?
g. How has it affected your relationships?
h. How has it changed the level or type of interpersonal conflict in your life?
i. How has it affected your home life?
j. How has it affected your physical health?
k. How has it affected your economic health?

(7) Would you refer other people to the treatment that you have received? Why or why not?

(8) What suggestions do you have to improve the treatment?

a. What other types of treatment would you like to receive?
b. What other settings/locations can treatment be provided?
c. Would you like to receive treatment by phone or in person? Why?
d. Were there any other areas/topics that you wanted to discuss with your treatment provider?

Conclusion

- Are there any other topics that you would like to discuss?
- Is there something you started to say about any topic that you did not get to share?
- Thank you very much for your time. Do you have any questions about this interview before we end today?
